# Supplementary material for: Antiviral Activity of Rhamnolipids Nano-Micelles Against Rhinoviruses—In Silico Docking, Molecular Dynamic Analysis and In-Vitro Studies
Source: Curr Issues Mol Biol. 2025 May 6;47(5):333. doi: 10.3390/cimb47050333 (PMC12110113; doi:10.3390/cimb47050333)
Supplement: Supplementary file 1 [file cimb-47-00333-s001.zip › cimb-3513043-supplementary.pdf]

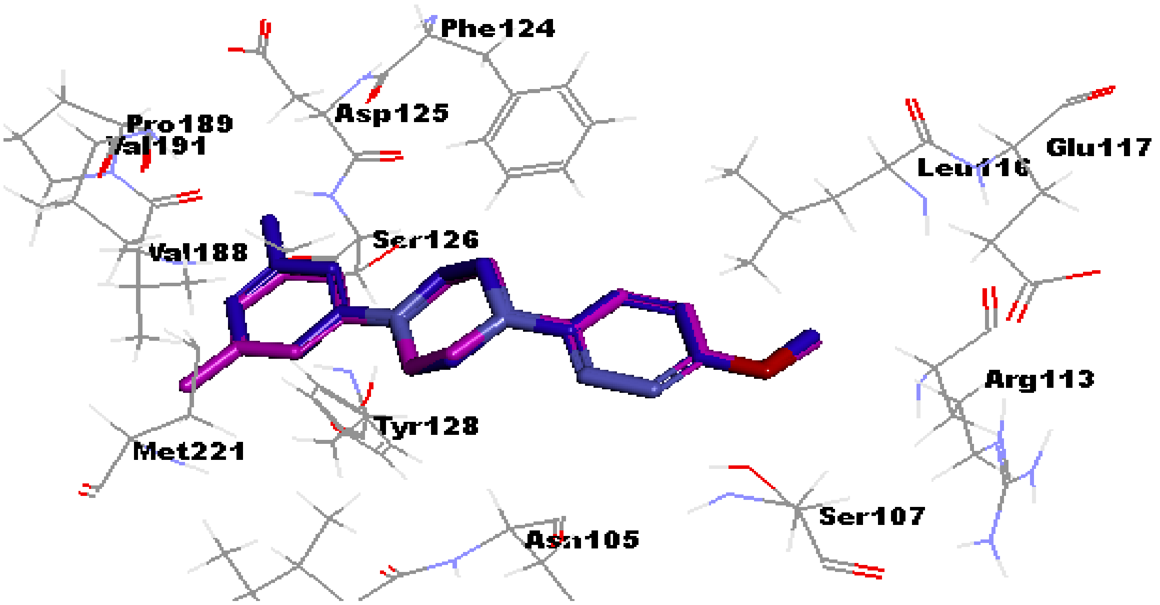

Figure S1: The alignment between the X-ray bioactive conformer of the co-crystalized antiviral R 61837 (colored in blue) and the docked pose of the same compound (colored in pink) at HRV-14 (1R09)binding site with RMSD value= 0.21A°.
